# DO PSYCHOSOCIAL RESOURCES CONFER PHYSIOLOGICAL RISK? EXAMINING ALLOSTATIC LOAD BLACK MEN ACROSS THE LIFE COURSE

**DOI:** 10.1093/geroni/igz038.2764

**Published:** 2019-11-08

**Authors:** Courtney S Thomas Tobin, Roland J Thorpe Jr

**Affiliations:** 1 UCLA Fielding School of Public Health, Los Angeles, California, United States; 2 Department of Health, Behavior, and Society, Baltimore, Maryland, United States

## Abstract

Research suggests positive psychosocial resources promote resilience, although this has been underexplored among Black men. The present study identified profiles of psychosocial resilience and examined their association with allostatic load (AL) among young, middle-aged, and older Black men. Data come from 283 Black men in the Nashville Stress and Health Study. Latent class analysis (LCA) identified resource profiles comprised of eight psychosocial resources across four categories (coping strategies, sense of control, racial identity, social support). Logistic regression was used to estimate the odds of high AL (5+ high-risk indicators) across classes. LCA indicated three latent classes: low (33%), moderate (26%), and high (41%) psychosocial resources. Unexpectedly, individuals in the high resilience class had the greatest relative odds of high AL; high resilience worsened health for older but not younger Black men. Findings suggest elevated levels of resources resilience may undermine physical health in this population.

